# Response letter: Examining the association between sleep apnea and total hippocampal volumes in cognitive impairment

**DOI:** 10.1002/alz.70602

**Published:** 2025-08-14

**Authors:** Matthew Settimi, Dennis Tchoudnovski, Ivan Ntale, David R. Colelli, Yakdehikandage S. Costa, Sara Mitchell, Mario Masellis, Benjamin Lam, Andrew Lim, Frances Chung, Joel Ramirez, Maged Goubran, Sandra E. Black, Mark I. Boulos

**Affiliations:** ^1^ Hurvitz Brain Sciences Research Program Sunnybrook Research Institute Sunnybrook Health Sciences Centre Toronto Ontario Canada; ^2^ Department of Medicine Division of Neurology University of Toronto Toronto Ontario Canada; ^3^ Undergraduate MD Program McMaster University Hamilton Ontario Canada; ^4^ Undergraduate MD Program University of Toronto Toronto Ontario Canada; ^5^ UCD School of Medicine University College Dublin Health Sciences Centre Dublin Ireland; ^6^ Undergraduate MD Program Memorial University St. John's, Newfoundland and Labrador St. John's Newfoundland Canada; ^7^ Department of Psychiatry University of Toronto Toronto Ontario Canada; ^8^ Sunnybrook Sleep Laboratory North York Ontario Canada; ^9^ Department of Anesthesia and Pain Management Toronto Western Hospital University Health Network University of Toronto Toronto Ontario Canada; ^10^ Dr. Sandra Black Centre for Brain Resilience and Recovery Sunnybrook Research Institute Sunnybrook Health Sciences Centre North York Ontario Canada; ^11^ Physical Sciences Platform Sunnybrook Research Institute Sunnybrook Health Sciences Centre North York Ontario Canada; ^12^ Department of Medical Biophysics University of Toronto Toronto Ontario Canada

1

Thank you, Dr. Xiao Le and Dr. Lin Fangbo, for your insightful comments. We have addressed the questions and incorporated your suggestions below.

Table 1 illustrates the demographics data of the excluded patients. While the two populations are quite similar, there are some notable demographic differences present. First, the average age of our excluded participants is higher. Additionally, previous stroke history appears more prevalent in the excluded cohort, regardless of obstructive sleep apnea (OSA) or cognitive status. Overall, our excluded participants tended to be slightly older, with smaller brain volumes, and a higher stroke prevalence. The excluded participants of each of the cognitive status groups had a larger percentage of patients with OSA, and subsequent increases in apnea–hypopnea index, time under 90% oxygen, and worsened mean SpO2. Evidently, these characteristic differences fall into the sequelae of older‐aged individuals.

**TABLE 1:** Demographic information of excluded participants stratified by type of cognitive impairment and OSA.

	Cognitive impairment of NVM etiology (*N* = 38)	Subjective cognitive complaints (*N* = 57)	OSA (*N* = 53)	No OSA (*N* = 39)
**Age in years, mean (SD)**	72 (10)	68 (11)	72 (9.8)	66 (11.0)
**Sex—male, % (N)**	46% (26)	46% (26)	63% (36)	26% (10)
**BMI, mean (SD)**	25 (4)	28 (6.4)	27 (4.9)	26 (6.9)
**Intracranial volume in mm^3^, mean (SD)**	1,197,287 (370,764)	1,340,332 (131,460)	1,330,005 (201,158)	1,319,415 (110,195)
**Total hippocampal volume in mm^3^, mean (SD)**	5928 (957)	5088 (1961)	5171 (1940)	5302 (1820)
**Years of education, mean (SD)**	15 (2.9)	15 (6)	15 (4.4)	16 (2.4)
**Hypertension, % (N)**:				
** None**	48% (27)	51% (20)	47% (23)	66% (19)
** Treated**	46% (26)	44% (17)	45% (22)	31% (9)
** Untreated**	5% (3)	5% (2)	8% (4)	3% (1)
**Diabetes, % (N)**:				
** None**	82% (46)	90% (35)	84% (41)	93% (27)
** Treated**	14% (8)	10% (4)	14% (7)	3% (1)
** Untreated**	4% (2)	0% (0)	2% (1)	3% (1)
**History of stroke, % (N)**	21% (12)	17% (6)	22% (10)	17% (5)
**OSA, % (N)**	64% (25)	56% (32)	–	–
**AHI, mean (SD)**	12 (12)	13 (17)	20 (17)	2.4 (2.3)
**T90%, mean (SD)**	20 (26)	2.6 (5.5)	12 (20)	5.9 (19)
**Mean SpO_2_, mean (SD)**	96 (1.8)	92 (13)	93 (1.7)	91 (19)
**Cognitive impairment of NVM etiology, % (N)**	–	–	56% (32)	64% (25)

Abbreviations: AHI, apnea–hypopnea index; BMI, body mass index; NVM, neurovascular, vascular, or mixed; OSA, obstructive sleep apnea; SD, standard deviation; SpO_2_, blood oxygen level; T90%, time under 90% oxygen.

One of the remaining differences between our excluded and analyzed patients that remain unexplained is that in the excluded participants analysis, for patients with OSA, we noted a smaller percentage of individuals who had concomitant cognitive impairment of neurovascular, vascular, or mixed (NVM) etiology (56% as opposed to 85%).

Ultimately, compared to Table 1 in our original article,^1^ we see many preserved relationships in the excluded patients as well.^1^ Patients with subjective cognitive complaints were younger, more educated, and relatively healthier (lower prevalence of hypertension and diabetes) compared to those with cognitive impairment of NVM etiology. Similarly, those with OSA were older and predominantly male, with worse sleep metrics. This is quite reassuring that our excluded participants were representative of our total analyzed population.

To assess whether associations hold across sexes, a linear regression model investigating interaction effects between OSA and male sex was performed. While both OSA and cognitive impairment of NVM etiology proved to be significantly associated with a decrease in hippocampal volume, the association with lower hippocampal volume did not appear to differ meaningfully between men and women when assessing the interaction between presence of OSA and male sex (β: 136.1 [95% confidence interval (CI): −363.6 to −635.8]; *P* = 0.591).

An additional linear regression model investigating the relationship between presence of OSA and total hippocampal volume was created, with two separate interaction terms: presence of OSA and male sex, and presence of OSA and NVM cognitive impairment. As expected, OSA and male sex was insignificant (β: 261.8 [95% CI: −232.8 to −756.3]; *P* = 0.297), while the interaction between OSA and NVM cognitive impairment reached significance (β: −889.9 [95% CI: −1483.7 to −296.1]; *P* = 0.004).

Finally, we ran linear regression analysis with a new, single variable coding each participant's cognitive impairment and OSA status (similar to the stratification shown in Table 3 of our publication).^1^ This analysis again yielded expected results: of the four possible cognitive impairment plus OSA status combinations, NVM+OSA was the only one that was significantly associated with total hippocampal volume (β: −802.2 [95% CI: −1338.8 to −265.6]; *P* = 0.004). In this analysis, there were no significant interaction effects between the cognitive impairment plus OSA status variable and male sex.

In the future, we would like to investigate the relationship between OSA and each of the four manifestations of cerebral small vessel disease on imaging: white matter hyperintensities, dilated perivascular spaces, lacunar infarcts, and cerebral microbleeds. Additionally, we are unable to perform threshold‐response analysis at this time; however, this is an excellent area for future exploration.

Unfortunately, the information regarding which scanner at Sunnybrook Health Sciences Center was used for each participant was not recorded when the data were initially collected. While we would like to update our models with the scanner type as an additional covariate, it is not feasible for us to do so at this time.

## AUTHOR CONTRIBUTIONS

Conceptualization: Matthew Settimi, Andrew Lim, Frances Chung, Maged Goubran, Sandra E. Black, and Mark I. Boulos. Critical revision of manuscript: David R. Colelli, Yakdehikandage S. Costa, Sara Mitchell, Mario Masellis, Benjamin Lam, Andrew Lim, Frances Chung, Joel Ramirez, Maged Goubran, and Sandra E. Black. Data acquisition: Matthew Settimi, David R. Colelli, Yakdehikandage S. Costa, Sara Mitchell, Mario Masellis, Benjamin Lam, Joel Ramirez, Sandra E. Black, and Mark I. Boulos. Data analysis and interpretation: Matthew Settimi and Mark I. Boulos. Interpretation and writing of the manuscript: Dennis Tchoudnovski and Ivan Ntale. Study design: Matthew Settimi, Andrew Lim, Frances Chung, Maged Goubran, Sandra E. Black, and Mark I. Boulos. Supervision of data acquisition: Joel Ramirez, Sandra E. Black, and Mark I. Boulos. Writing of the manuscript: Matthew Settimi, Dennis Tchoudnovski, Ivan Ntale, and Mark I. Boulos.

## CONFLICT OF INTEREST STATEMENT

M.S., D.T., I.N., D.R.C., Y.S.C., B.L., and J.R. do not have any conflicts of interest. Non‐financial support from S.M. outside of the submitted work, Dr. Mitchell perceived the following conflicts/disclosures unrelated to this manuscript: Dr. Mitchell receives philanthropic support from the Azrieli Foundation, P. Austin Family Foundation, and Great Gulf Foundation. She has received consulting fees from Eli Lilly Canada, Eisai Canada, and Novo‐Nordisk Canada. Non‐financial support from M.M. outside of the submitted work, Dr. Masellis perceived the following conflicts/disclosures unrelated to this manuscript. Dr. Masellis holds grants to the institution from the Canadian Institutes of Health Research, Washington University, Weston Brain Institute, Women's Brain Health Initiative, and Brain Canada. He has funding for contract research from Roche and Alector. He receives royalties from Henry Stewart Talks. He has received consulting fees from Eli Lilly Canada, Alector, Biogen Canada, Wave Life Sciences, Eisai Canada, and Novo‐Nordisk Canada. He has received speaker honoraria from MINT Memory Clinics and the ECHO Dementia Series. He has sat on scientific advisory boards for Alzheimer Society Canada and Parkinson Canada; these are unpaid roles. Non‐financial support from A.L. outside of the submitted work, Dr. Andrew Lim reports funding from the Canadian Institutes of Health Research, National Institutes of Health, and Weston Foundation. F.C. received research support from the Ontario Ministry of Health and Long‐Term Care Innovation Grant, ResMed Foundation, University Health Network Foundation. Non‐financial support from M.G. outside of the submitted work, Dr. Maged Goubran reports funding from CIHR, NSERC, NIH, CFI, Canada Research Chairs program, Alzheimer's Society, Alzheimer's Association, Digital Research Alliance of Canada, and the Harquail Centre for Neuromodulation. Non‐financial support from S.E.B. outside of the submitted work, Dr. Sandra Black reports funding made to the institution for contract research (no personal investigator fees taken) from Genentech, Optina, Roche, Eli Lilly, Eisa/Biogen Idec, NovoNordisk, Lilly Avid, ICON, Aribio Co. She received peer reviewed funding made to the institution (no personal investigator fees taken) from Ontario Brain Institute, CIHR, Leducq Foundation, Heart and Stroke Foundation of Canada, NIH, Alzheimer's Drug Discovery Foundation, Brain Canada, Weston Brain Institute, Canadian Partnership for Stroke Recovery, Canadian Foundation for Innovation, Focused Ultrasound Foundation, Alzheimer's Association US, Queen's University, Compute Canada Resources for Research Groups, CANARIE, Networks of Centres of Excellence of Canada. She received consulting fees and honoraria from Roche, Biogen, NovoNordisk, Eisai Canada, Eli Lilly, and Livagency. She has also participated in advisory boards for the Conference Board of Canada, World Dementia Council, National Institute of Neurological Disorders and Stroke, and Ontario Dementia Care Alliance (ODCA). Non‐financial support from M.I.B. outside of the submitted work, Dr. Mark Boulos reports funding from the Canadian Institutes of Health Research, Heart and Stroke Foundation of Canada, CanStroke Recovery Trials network, Restless Legs Syndrome Foundation, Alternative Funding Plan from the Academic Health Sciences Centres of Ontario, the Temerty Centre for AI Research and Education in Medicine, and the McLaughlin Centre for Molecular Medicine. He also reports consulting fees and honoraria from Jazz Pharmaceuticals, Paladin Labs, Eisai, and the OntarioMD Peer Leader Program; travel support from McGill University; and receipt of sleep equipment or research support from Braebon Medical Corporation, CGX, The Mahaffy Family Research Fund, and Green Mountain. Author disclosures are available in the Supporting information.

## Supporting information



Supporting Information
